# Patient and Public Involvement in Youth Mental Health Research: Protocol for a Systematic Review of Practices and Impact

**DOI:** 10.3389/fpsyg.2021.703624

**Published:** 2021-11-05

**Authors:** Célia M. D. Sales, Filipa Martins, Marisa M. Alves, Sara Carletto, Sonia Conejo-Cerón, Luis Costa da Silva, Anja Čuš, Chloe Edridge, Nuno Ferreira, Camellia Hancheva, Esperanca M. A. Lima, Shaun Liverpool, Nick Midgley, Bettina Moltrecht, Patricia Moreno-Peral, Nicholas Morgan, Rose Mortimer, Catarina Pinheiro Mota, Giada Pietrabissa, Sonia Sousa, Randi Ulberg, Julian Edbrooke-Childs

**Affiliations:** ^1^Faculty of Psychology and Education Science, University of Porto, Porto, Portugal; ^2^Center for Psychology, University of Porto (CPUP), Porto, Portugal; ^3^Department of Neuroscience “Rita Levi Montalcini”, University of Torino, Turin, Italy; ^4^Biomedical Research Institute of Malaga (IBIMA), Málaga, Spain; ^5^Evidence Based Practice Unit, University College London and Anna Freud National Centre for Children and Families, Clinical, Educational and Health Psychology, London, United Kingdom; ^6^Child Outcomes Research Consortium, Anna Freud National Centre for Children and Families, London, United Kingdom; ^7^Department of Child and Adolescent Psychiatry, Medical University of Vienna, Vienna, Austria; ^8^Department of Social Sciences, University of Nicosia, Nicosia, Cyprus; ^9^Sofia University St. Kliment Ohridski, Sofia, Bulgaria; ^10^Faculty of Health, Social Care and Medicine, Edge Hill University, Ormskirk, United Kingdom; ^11^University of Trás-os-Montes and Alto Douro, Vila Real, Portugal; ^12^Department of Psychology, Catholic University of Milan, Milan, Italy; ^13^Istituto Auxologico Italiano IRCCS, Psychology Research Laboratory, Milan, Italy; ^14^School of Digital Technologies, Tallinn University, Tallinn, Estonia; ^15^Division of Mental Health and Addiction, Institute of Clinical Medicine, University of Oslo, Oslo, Norway; ^16^Department of Psychiatry at Diakonhjemmet Hospital, Oslo, Norway

**Keywords:** patient and public involvement, involvement in research, adolescents, young people, mental health

## Abstract

Various health settings have advocated for involving patients and members of the public (PPI) in research as a means to increase quality and relevance of the produced knowledge. However, youth PPI has been an understudied area. This protocol paper describes a new project that aims to summarize what is known about PPI with young people in mental health research. In line with the Preferred Reporting Items for Systematic reviews and Meta-Analyses Statement guidelines we will identify and appraise suitable articles and extract and synthesize relevant information including at least two reviewers at each stage of the process. Results will be presented in two systematic reviews that will describe (a) how youth PPI has been conducted (Review1) and (b) what impact youth PPI had on the subsequent research and on stakeholders (Review2). To our knowledge, this is the first set of reviews that uses a critical appraisal tool, which is co-developed with children and young people. Findings from this project will provide valuable insights and set out the key steps to adopting adequate PPI methods when involving children and young people in mental health research.

## Introduction

Over the last two decades, there has been an increasing international recognition of the importance of involving patients and the public in health research (scientific projects aimed at increasing knowledge) (McCoy et al., 2019). Patient and public involvement (PPI) can be defined as “research being carried out ‘with’ or ‘by’ members of the public rather than ‘to,’ ‘about’ or ‘for’ them.” (INVOLVE, n.d.). The term “patients and public” refers to people who are “experts” on the researched topic because of their experiential knowledge, as “patients, potential patients, caregivers, and people who use health and social care services, as well as people from organizations that represent people who use services” (p.6) (Hayes et al., 2012). It also refers to members of the general public, “lay representatives” who contribute to knowledge development with a distinctive perspective to that of researchers or health professionals (Wilson et al., 2015). PPI assumes a post-constructivist epistemological orientation that highlights the importance of subjective experiences in knowledge construction (Minkler and Wallerstein, 2008). It denotes a new way of producing science where experts by experience take an active role as co-researchers in different phases of the research cycle, which may include designing, delivering the research and disseminating its results.

Patient and public involvement in health research has the potential to increase the relevance of the scientific knowledge produced, through identifying research questions and prioritizing research agendas, designing more appropriate and meaningful approaches to conducting the research, addressing ethical tensions, and matching research with policy objectives (Brett et al., 2014a, b; Mitchell et al., 2019). Final reports or publications benefit from being grounded in user experiences and provide a wider and more relevant viewpoint, by ensuring cultural relevance and by giving the results better credibility with stakeholders (Brett et al., 2014a, b). Dissemination and implementation of the research findings also benefit from PPI because the public can relate to the findings of their own experiences and present them in a more user-friendly way (Brett et al., 2014a, b). Moreover, the interest of promoting patient-centered research has been reinforced through the integration of PPI with the ethical argument for involvement in research (Delbanco et al., 2001; Wilson et al., 2015). Politically PPI is considered an opportunity for addressing the democratic deficit by giving voice to the public in publicly funded health organizations and research (Boivin et al., 2010). Adolescents and young people in particular can be valuable partners in research, by giving their unique views on what and how research should be done, or by assuming an active role in research tasks, such as recruitment of peer participants, data collection, data analysis, participation in dissemination materials, among others (e.g., Coad and Evans, 2008; Mawn et al., 2015). Youth PPI can benefit not only research, but also the young people involved and the professionals (van Schelven et al., 2020). Because of these expected benefits, there is a general consensus among health research agencies that PPI should be a standard element of research projects.

However, there has been criticisms pointing to the limited evidence on the real (and not only expected) impacts of PPI (the resulting effects of undertaking PPI in a research study) (e.g., Bailey et al., 2015). Such lack of evidence of PPI has been associated to inadequate or insufficient reporting of the practices and the absence of methods to assess impacts (Staniszewska and Denegri, 2013; van Schelven et al., 2020; Gjoneska et al., 2021; Jones et al., 2021). Many studies include only partial information, which hinders our understanding of what works, for whom, in what context and why. In order to validate PPI approach and to identify the most effective forms of PPI in particular settings it is necessary a critical appraisal of the literature, using existing knowledge (e.g., systematic reviews) as a starting point to address PPI challenges (Staniszewska and Denegri, 2013; van Schelven et al., 2020). This is more important in understudied areas as youth PPI, which presents particular challenges. For instance, young people have dynamic lives, balancing education, sport, social activities, part-time employment, etc. Fast lifestyle and developmental changes take place, resulting in fluctuation or low adherence along the research process (Mawn et al., 2015). Some studies have also described a risk of dropping out due to young people losing interest, or being afraid of stigmatizing or losing respect from peer groups (van Schelven et al., 2020).

This protocol presents a project that aims to systematically describe the landscape of the understudied area of youth PPI in mental health research. Young people have been particularly excepted from the process of influencing mental health research. In most studies their perspectives are ignored or filtered through the interpretations of adult researchers or their carers (Mawn et al., 2015). To the best of our knowledge, the present review is the first to systematically address mental health and psychotherapy research specifically. Previous reviews on youth PPI in health research have focused mainly on chronic health condition management, lifestyle advice, or involvement of disabled children and young people (Bailey et al., 2015; Larsson et al., 2018; van Schelven et al., 2020). However, mental health research involving children and young people requires particular guidance.

Our objective is to map youth PPI in mental health research, conducting two interrelated systematic reviews addressing two overarching research questions: (1) how youth PPI in mental health research has been implemented, as well as the demographics and lived experience characteristics of young people most frequently involved in PPI, in order to explore whether there are groups over- and under-represented in PPI of youth mental health research (Review 1); and (2) What are the impacts of youth PPI in mental health research (Review 2). Our specific research questions are:

Review 1:

1. What approaches are used for PPI in mental health research with young people?
2. What groups of young people (11–20 years) are most frequently involved in PPI in mental health research?
3. To what extent is PPI in mental health research with young people (11–20 years) reported according to recommended guidelines?
4. What are young people’s (11–20 years) experiences of PPI in mental health research?
5. What are the young person-reported and researcher-reported barriers and facilitators to PPI in mental health research with young people (11–20 years)?

Review 2:

1. What are the reported impacts of PPI in mental health research with young people?
2. What aspects of PPI context and process are associated with its positive and negative impacts in mental health research with young people?
3. How have impacts of PPI in mental health research with young people been assessed?

## Methods

### Study Design

The studies adopt a systematic review methodology, both attending to the principles provided by the Preferred Reporting Items for Systematic reviews and Meta-Analyses Statement guidance (Page et al., 2021). The systematic reviews have been registered with the PROSPERO (International Prospective Register of Systematic Reviews) database (registration numbers: CRDCRD42020171476 and CRD42021224682). The reviews are part of the work being developed by the COST Action TREATme, which aims to improve knowledge and understanding of psychotherapeutic interventions in young people.

### Note on the Research Process

Review 1 and 2 propose to answer different research questions regarding studies that share the same characteristics. Hence, as eligibility criteria for both reviews is shared, the systematic processes of screening for eligible papers served both reviews. For extracting the data, the research team will develop an extraction spreadsheet that includes the common and specific categories from both reviews, so that the data extraction for both studies could be concurrently performed.

### Data Sources

Searched databases comprised PsycINFO (OVID), MEDLINE (OVID), EMBASE (OVID), Web of Science core collection, Current Contents Connect, SciELO Citation Index, Cochrane Library of Systematic Reviews, CINAHL (EBSCO), ERIC (EBSCO), and child and adolescent studies (EBSCO). Systematic searches were undertaken to identify records encompassing the period from January 2000 to January 2020.

Two researchers (JEC, LCS/FM) have independently performed the searches. For each database, a search strategy was developed, comprising three concepts: children and young people (participants), mental health (condition), and patient and public involvement (intervention), informed by previous reviews (Crocker et al., 2018). As recommended, the search strategy was recorded in PROSPERO prior to the independent searches (Martin et al., 2020, 2021). Citation tracking of included papers was performed, and retrieved hits were exported to EndNote and Excel for title and abstract screening.

### Eligibility Criteria

Included papers will be in the domain of mental-health research, comprising studies on mental-health intervention or psychotherapy. As our focus is on involving young people with lived experience of mental health difficulties in mental health research, we will include studies in which young people with experience of mental health difficulty or accessing mental health support were involved in PPI. Therefore, studies on mental-health prevention, which in our scoping stage typically involved young people who did not necessarily have such lived experience of mental health difficulties, will be excluded. Eligible studies will involve PPI with young people aged between 11 and 20 years old. Regarding study design, all types will be eligible for inclusion (e.g., controlled trial, uncontrolled trial, pre-post study, cross-sectional study, pilot/feasibility trial, qualitative study, mix-methods study, methodological study, developmental studies study, or others), except for case studies or case series. The inclusion/exclusion criteria are shown in Table 1.

**TABLE 1:** Inclusion/Exclusion criteria.

	Include	Exclude
Sample	Studies targeting young people of a mean age between 11 and 20 years experiencing mental health difficulties, accessing psychotherapy and/or using other mental health interventions.	Studies targeting younger children (<11 years) or older young people (>20 years) and focused on prevention interventions.
Phenomenon of Interest	Studies with describing an element of Patient and public involvement (PPI).	
Study Design and Research Type	Studies with qualitative, quantitative or PPI centered data from any of the following: controlled or uncontrolled studies; pre-post studies; cross sectional studies; methodological or developmental studies; pilot/feasibility trials or reflections from the field.	Studies with insufficient information for data extraction and case study/series design.
Evaluation	Studies describing: approaches used for PPI; groups involved in PPI; extent of PPI reporting; young people’s and researcher’s experience of PPI; and the contexts/processes facilitating impact of PPI.	

*PPI = patient and public involvement.*

### Study Selection and Data Extraction

Two independent reviewers will perform title and abstract screening, with one reviewer (FM) conducting all title and abstract screening and a second screening being equally distributed among the remaining research team. All full-text screening will be conducted by three reviewers (FM, EL, MA) and a second screening will be equally distributed among the remaining researchers. A piloting of 15 papers will be done, for both title and abstract screening and full-text screening stages. Data extraction and quality assessment will be performed by one reviewer (FM) and cross-checking of all data extraction will be equally distributed among remaining researchers. A pilot of three papers will be conducted for data extraction and quality assessment.

Information from each study will be extracted for the following categories: author; year; title; citation; country; primary aim(s) of research; research participants setting; research participants sociodemographics; study design; intervention; comparator; young researchers setting; young researchers sociodemographics; young researchers lived experiences; results of PPI activities; impacts of PPI; methods and processes used to assess impacts and outcomes of PPI; PPY type; PPI content, format and stage; PPI sessions number; provided training to young people; provided support to young people; PPI theory/framework; attitudes toward PPI of the people involved; relationships and communication between people involved; planned time needed for PPI activities; planned funding of PPI activity; young people’s experience/feedback of PPI; researchers’ experience/feedback on PPI; young person-reported barriers and facilitators to PPI; researcher-reported barriers and facilitators to PPI; ethical approval for PPI; written informed consent for PPI.

### Critical Appraisal

Given the aims of the present review and the heterogeneity of the considered study designs, a critical appraisal of bias assessment of study quality is deemed not appropriate or feasible to conduct with existing tools. Therefore, the main focus of critical appraisal will cover the quality and completeness of reporting of essential elements of PPI to increase transparency and reproducibility. Correspondingly, we worked with young people with lived experience of mental health difficulties to review existing guidelines (Staniszewska et al., 2017) and co-produced Reporting Guidelines for PPI in mental health research with young people: Design through to delivery (please see Supplementary Appendix 1). Using these guidelines, each of the included studies will be rated by two independent reviewers to assess the quality and quantity of reporting of PPI.

### Data Synthesis

Given the aims of the present review, a meta-analysis will not be performed. Instead, we will conduct a meta-synthesis of the narrative findings. This will involve carefully reading and re-reading each study, line-by-line coding of the manuscript by at least two different reviewers, grouping the codes into a hierarchical structure, and generating analytical themes (Brett et al., 2014b). For review 1, a descriptive summary of approaches used for PPI will be presented, and a thematic synthesis (Barnett-Page and Thomas, 2009) will be conducted to identify types of approaches and their similarities and differences. Similarly, a descriptive analysis of the lived experience (e.g., presenting problem) of young people involved in PPI will be conducted. The extent to which each study reports PPI in line with recommended guidelines will be analyzed using a descriptive summary, which will be charted using the co-produced reporting guidelines. A thematic synthesis will be conducted to identify themes of young people’s experiences of PPI. A thematic synthesis will also be conducted to identify themes of barriers and facilitators to PPI as reported by young people and researchers.

For review 2, a qualitative narrative synthesis of the data will be undertaken, through familiarization with the papers and the identification of themes. Data synthesis will follow the framework of analysis used for the PIRICOM systematic review (Brett et al., 2010), a framework that has informed other reviews on the impacts and outcomes of PPI (Brett et al., 2014a, b). The PIRICOM systematic review proposed a synthesis in which both beneficial and challenging impacts of PPI on health and social care are reported. Such impacts are categorized as: impacts on the research and the research processes, impacts on end-users, researchers, research participants, the community, journals, policy makers, and funders. This framework of analysis includes reporting on PPI outcomes, which are presented according to the following categories: agenda setting; ethical decisions; methodology and data collection; writing up and dissemination; dissemination and implementation of results; and when users are involved in most stages. The PIRICOM systematic review also emphasizes the importance of considering contexts and processes of PPI when discussing its impacts. Contexts and processes of PPI are the set of factors that need to be in place in order to enable PPI to have an impact. Contexts may include the environment in which PPI is undertaken (e.g.: funding, policy, attitudes), while processes refer to the structure of PPI (e.g.: level of engagement, stages of involvement). The PIRICOM systematic review acknowledges that most studies do not report in detail the contexts and processes of PPI, however, the identification of some of these factors and a more general discussion on how they are linked to PPI impacts are included in its framework of analysis and will accordingly be adopted for the synthesis of review 2.

### Patient and Public Involvement

As previously stated, young people were involved in the co-creation of the reporting guidelines that will guide the critical appraisal of the studies.

### Ethics and Dissemination

As this is a secondary data analysis no ethical approval will be necessary to conduct the review. Dissemination will be done *via* peer-reviewed open access journal publications, conferences, seminars, and through the COST TREATme Action homepage. Additionally, the search data set will be published in an open data repository after the acceptance for the publication of the reviews in order to facilitate access to students, academics, and professionals.

## Discussion

This proposed review will add to the literature in several ways. To our knowledge, this may be the first set of reviews to use a critical appraisal tool developed in collaboration with young people. Previous reviews have used standardized critical appraisal tools which may not reflect components that are important to young people involved in research. Further, the impact of this review is the proposed synthesis of data for the provision of evidence-based PPI. Other important eventual protocol amendments that enhance our knowledge of PPI will be documented and noted in the future discussion.

The importance of involving young people in mental health research has been recognized by several countries (Brett et al., 2014a, b). Thus, this review may give an overview of the current practices and impact of PPI on young people in mental health research. With this knowledge we may also provide an insight into what impact of PPI with young people is commonly reported and how it is assessed. This twofolded review will hence give an overview of how and when young people were included as active partners in research on mental health, what are the impacts of such partnerships, and what barriers and facilitators to this process were identified by the research teams and young people.

The outcomes of the two reviews may be relevant to facilitate and inform future active partnerships among young people, mental health professionals, researchers, and decision makers. Importantly, by learning how PPI is currently organized and what information is provided in research studies, further PPI studies may result in improved practices and reporting. In the same vein, this study could encourage stakeholders to share lessons learnt during research collaborations with young people. While valuing the subjective and unique knowledge of young people with first-hand experience of mental health difficulties, in line with a post-constructivist approach in science, this review will add a critical view of the contributions of youth PPI to knowledge creation in the mental health research panorama.

Due to the scarcity of PPI literature as a whole, and even less so in young people mental health, it is expected that there will be relatively few publications examining such approaches directly. Therefore, this review has designed a thorough search strategy that will encapsulate as many relevant publications as possible. However, a potential limitation of conducting two reviews simultaneously is the possible time lag involved. For example, while registration for PROSPERO occurs fairly quickly, the steps involved for submission, review, and eventual publication of the study protocol article, and the actual systematic reviews, will most likely take several months each. Potential methodological limitations in this systematic review include a wide heterogeneity in the mental health problems studied, in the types of designs used, and in the mental health interventions carried out. The experiences and the barriers and facilitators reported by young people of PPI in mental health research could be different depending on the type of intervention or condition being studied. However, the use of broad inclusion criteria will allow us to describe the existing knowledge comprehensively and increase external validity of our conclusion.

## Author Contributions

CS and JE-C conceptualized the study. JE-C, FM, and LS carried out the database searches. All authors were involved in the selection of articles, data extraction and analysis, writing and editing of the original draft, and approved the final submission.

## Conflict of Interest

The authors declare that the research was conducted in the absence of any commercial or financial relationships that could be construed as a potential conflict of interest.

## Publisher’s Note

All claims expressed in this article are solely those of the authors and do not necessarily represent those of their affiliated organizations, or those of the publisher, the editors and the reviewers. Any product that may be evaluated in this article, or claim that may be made by its manufacturer, is not guaranteed or endorsed by the publisher.
