# Supplementary material for: Patient and Public Involvement in Youth Mental Health Research: Protocol for a Systematic Review of Practices and Impact
Source: Front Psychol. 2021 Nov 5;12:703624. doi: 10.3389/fpsyg.2021.703624 (PMC8603822; doi:10.3389/fpsyg.2021.703624)
Supplement: Supplementary file 1 [file Table_1.DOCX]

###

**Reporting guidelines for Patient and Public Involvement (PPI) in mental health research with young people: Design through to delivery**

| Criteria | Reported (i.e., Yes, No, Unclear | Evidence (i.e., Page, Line) |
| --- | --- | --- |
| 1. The concept of Patient and Public Involvement or co-design is mentioned in the title and/or abstract. |  |  |
| 1. The objective of the PPI is clearly described. |  |  |
| 1. Involvement of young people is embedded in the project design and methodology. |  |  |
| 1. There is clear reporting of which stage in the research process the PPI has taken place for example differentiating between involvement in the design and planning of prioritising and commissioning, evaluation or dissemination |  |  |
| 1. The recruitment method including eligibility criteria for involvement is described comprehensively. |  |  |
| 1. Where possible, the recruitment procedure allows for a involvement of a representative group, including demographics (e.g., age, gender, ethnicity, sexual orientation) and type of experience (e.g., direct and indirect experience of the subject matter). |  |  |
| 1. Individual support requirements were considered on an ongoing basis and any actions or decisions relating to this described; for example, relating to individuals’ age, mental health and access needs. |  |  |
| 1. Clear goals, guidelines and expectations for the PPI were agreed mutually, and are reported. The process for reaching agreement may also be reported. |  |  |
| 1. It is reported how participation in the PPI was made as accessible as possible for young people, for example, removing psychological and research “jargon” where appropriate. |  |  |
| 1. A description of the setting and overall atmosphere is given, and reasons for the choice of setting are detailed. For example, a description of the physical space or online platform. |  |  |
| 1. A comprehensive description is given of the participation activity/activities. |  |  |
| 1. It is reported how actioning feedback from those involved was considered. |  |  |
| 1. The outcomes of the PPI are comprehensively described, and if there has been more than one participation, element it is clearly presented which outcomes relate to the different participation stages. |  |  |
| 1. Challenges that occurred are reported, as are the methods that were used to overcome them. |  |  |
| 1. The reporting describes how those involved have been thanked for their involvement and appropriately paid or reimbursed (e.g., for time and travel). Young people may also be given the opportunity to contribute to publications or other communications, and if so, this is clearly acknowledged in authorship. |  |  |
| 1. Plans to keep young people updated with the outcome and impact of their involvement are reported. |  |  |

Please cite as Edridge, C., Morgan, N., Mortimer, R., Anna Freud Centre Young Champions, Sales, C., Martins, F., Alves, M., Carletto, S., Conejo-Cerón, S, Costa da Silva, L., Čuš, A., Ferreira, N., Hancheva, C., Lima, E., Liverpool, S., Midgley, N., Moltrecht, B., Moreno-Peral, P., Morgan, N., Mota, C. P., Pietrabissa, G., Sousa, S., Ulberg, R., and Edbrooke-Childs, J. (2020). *Reporting guidelines for Patient and Public Involvement (PPI) in mental health research with young people: Design through to delivery*. London: EBPU.

This project was supported by TREATme, a European Network on Individualized Psychotherapy Treatment of Young People with Mental Health Disorders under the European Cooperation in Science and Technology which is supported by the EU Framework Programme Horizon 2020 action CA16102.

Last updated: March 2021
